# Cerebrospinal Fluid and Brain Tissue Penetration of Tenofovir, Lamivudine, and Efavirenz in Postmortem Tissues with Cryptococcal Meningitis

**DOI:** 10.1111/cts.12661

**Published:** 2019-07-10

**Authors:** Melanie R. Nicol, Katelyn A. Pastick, Joneé Taylor, Olivie C. Namuju, Joshua Rhein, Darlisha A. Williams, David B. Meya, David R. Boulware, Robert Lukande

**Affiliations:** ^1^ Department of Experimental and Clinical Pharmacology College of Pharmacy University of Minnesota Minneapolis Minnesota USA; ^2^ Department of Medicine Medical School University of Minnesota Minneapolis Minnesota USA; ^3^ New York City Office of the Chief Medical Examiner and New York University New York City New York USA; ^4^ Infectious Disease Institute of Makerere University Kampala Uganda; ^5^ Department of Pathology Makerere University Kampala Uganda

## Abstract

The central nervous system (CNS) is a known HIV reservoir, yet little is known about drug exposure in the brain. Our primary objective was to quantify exposure of three common antiretrovirals in brain tissue and compare exposures to plasma and cerebrospinal fluid (CSF). We also sought to identify pockets of brain most vulnerable to inadequate drug exposures and examine the role of meningitis in drug penetration into the CNS. Tenofovir, lamivudine, and efavirenz concentrations were measured using liquid chromatography and tandem mass spectrometry in plasma and CSF from 14 individuals with HIV, 7 with cryptococcal meningitis. In four individuals (three with meningitis) drug concentrations were also measured in 13 distinct brain tissue regions. In subjects with meningitis, geometric mean ratio (95% confidence interval) of tenofovir CSF to plasma was 66% (7–598%) and 14% (6–31%) in subjects without meningitis. Lamivudine CSF penetration was 100% (25–409%) in subjects with meningitis and 30% (24–37%) in subjects without meningitis. Tenofovir brain tissue concentrations were 36% (14–124%) of plasma and 49% (1–572%) of CSF. Lamivudine brain concentrations were 37% (23–64%) of plasma and 27% (1–104%) of CSF. Efavirenz brain tissue concentrations were 128% (108–179%) of plasma. Tissues collected postmortem provide a unique opportunity to assess drug distribution in tissues difficult to sample in living subjects. CSF is a poor surrogate for drug exposure throughout the CNS. Antiretrovirals differentially penetrate into the CNS and penetration may be enhanced by meningitis.

Despite HIV therapy, persistent viral replication occurs in certain anatomic sites, and systemic viral rebound will occur within weeks of treatment interruption.^1^ The central nervous system (CNS) is hypothesized to be a potential HIV reservoir, harboring virus that is either resistant or not exposed to adequate antiretroviral therapy.^2^ CNS Penetration‐Effectiveness scores, largely based on drug exposure in cerebrospinal fluid (CSF), were developed for antiretrovirals to provide a prescribers guidance on which regimens have greatest likelihood of achieving therapeutic CNS concentrations.^3^ However, brain extracellular fluid can either be passively secreted to CSF or actively transported into arterial blood.^4^ Therefore, CSF may not represent drug exposure at the CNS sites where pathogens preside, where drug action is most relevant.

Inflammation at the blood–CSF barrier or the CSF–brain barrier could lead to altered drug distribution via opening of tight junctions, altered pH changing drug ionization, and downregulation of efflux transporters.^5^ The effect of meningitis coinfection on antiretroviral CNS penetration has not previously been quantified.

Postmortem drug quantification has been used in forensics for decades to assist with determining cause of death, in particular when overdose was suspected. Here, we describe the use of brain specimens collected during autopsy to quantify drug exposure of three commonly used antiretrovirals: tenofovir, lamivudine, and efavirenz. We sought to compare exposures to plasma and CSF and to identify pockets of brain most vulnerable to inadequate drug exposures. We also examined the effects of meningitis on drug penetration into CSF.

## Methods

### Sample collection

Postmortems were performed in Kampala, Uganda, from 2017–2018 on a cohort of Ugandans with known HIV. Written informed consent was obtained from next of kin under a protocol approved by the Research Ethics Committee of Mulago National Referral Hospital. Time of last dose and time of death were collected by hospital records or caregiver interview. During autopsy, tissue sections (1 g) from discrete brain regions were briefly rinsed with normal saline to remove blood and immediately snap frozen in liquid nitrogen. Whole blood was collected from the femoral vein into EDTA tubes and plasma separated. CSF was collected via cisternal puncture. Samples were stored at −80°C.

### Analytical methods

Prior to drug quantification, ~ 0.3 g of brain tissue was combined with twice the volume (0.6 mL) of 5% bovine serum albumin. Sample was homogenized with Tissue Tearor (BIOSPEC Products) and centrifuged for 15 minutes at 15,000*g*. The top supernatant layer was removed for sample extraction. Internal standards (tenofovir‐d6, lamivudine C13, and efavirenz d‐4) were added to the samples. Sample proteins were precipitated with cold acetonitrile. Detection and quantification of tenofovir and lamivudine was performed in all matrices using an Acquity ultrahigh‐performance liquid chromatograph (Waters, Milford, MA) coupled with a Quattro Ultima triple state quadrapole mass spectrometer (Micromass, Manchester, UK). Efavirenz assay was performed using a high‐performance chromatograph (Agilent 1200 Series, Santa Clara, CA) coupled with a TSQ Quantum triple stage quadrupole mass spectrometer (Thermo‐Electron, San Jose, CA). Calibrator standards and quality control samples were prepared in the matrix and frozen in advance to match the sample tested; bovine brain homogenate was used for brain tissue, a solution of salt and proteins for CSF, and lithium heparin for plasma. Following a 3‐day validation, tenofovir accuracy was 94%, 104%, and 106% in plasma, CSF, and brain, respectively, whereas lamivudine was 95%, 94%, and 95%. Precision was 6.6%, 5.5%, and 5.8% for tenofovir and 2.5%, 7.1%, and 2.8% for lamivudine in plasma, CSF, and brain. Efavirenz accuracy in plasma was 84% with 3% variability; in brain tissue, accuracy was 93% with 4.5% variability. Efavirenz in CSF was not quantified due to low solubility of efavirenz in CSF solution and expected low concentrations of efavirenz in CSF.

Plasma creatinine and CSF/plasma albumin ratio (AR)were also quantified by Fairview Diagnostics Laboratory, a CAP‐accredited and CLIA‐accredited clinical laboratory in St. Paul, Minnesota (https://www.fairview.org/services/laboratory-services/fairview-diagnostic-laboratories).

### Statistical methods

Data are reported as median (25th and 75th percentile) or geometric mean ratio (GMR; 95% confidence interval) assuming a tissue density of 1 g/mL. Mean brain tissue concentrations were calculated for each individual by averaging all brain tissues (except CSF). Spearman rank correlation test was used to test for correlations between variables. Differences between compartments were tested using logarithmic paired *t*‐test. Wilcoxon rank sum test was used for comparisons by meningitis status. Statistics were performed in SAS version 9.4 (Cary, NC).

## Results

### Plasma and CSF concentrations associated with creatinine but not postmortem interval

Plasma and CSF were collected from 16 individuals with HIV (**Table** **S1**); 14 were on antiretrovirals at the time of death. In the 11 individuals receiving tenofovir, median (25th and 75th percentile) plasma concentrations were 1,024 (247–2,683) ng/mL, whereas CSF concentrations were 138 (77–675) ng/mL. Tenofovir plasma and CSF were highly correlated (*r* = 0.77, *P* = 0.005). Lamivudine plasma concentrations in 14 individuals were 1,315 (657–4,522) ng/mL. Lamivudine CSF concentrations were 566 (366–1,638) ng/mL and highly correlated with plasma (*r* = 0.82, *P* < 0.001). Efavirenz plasma concentrations in 11 individuals were 3,342 (310–4,860) ng/mL. The time between death and tissue collection was not correlated with drug concentration in any matrix (**Table** 1). The time between last reported dose and tissue collection was correlated with lamivudine plasma and CSF concentrations but not tenofovir or efavirenz concentrations in either matrix (**Table** 1).

**Table 1:** **Correlation tests between drug concentrations and other variables**

	*n*	Plasma creatinine		Hours between death and tissue collection		Hours between last recorded dose and tissue collection	
		Spearman coefficient	*P* value	Spearman coefficient	*P* value	Spearman coefficient	*P* value
Tenofovir plasma	11	0.74	0.0098	0.24	0.474	−0.14	0.760
Tenofovir CSF	11	0.74	0.0098	0.51	0.113	−0.21	0.645
Lamivudine plasma	14	0.62	0.018	0.21	0.463	−0.70	0.025
Lamivudine CSF	14	0.59	0.027	0.03	0.917	−0.75	0.013
Efavirenz plasma	11	−0.15	0.650	−0.30	0.369	−0.14	0.760

CSF, cerebrospinal fluid.

Creatinine was measured to estimate kidney function at the time of death. Plasma creatinine was not associated with postmortem interval (*r* = 0.02, *P* = 0.9). Plasma creatinine was significantly correlated with tenofovir and lamivudine concentrations but not efavirenz concentrations (**Table** 1). When cases with creatinine clearance <30 mL/min were excluded, median (range) plasma concentrations were 212 (134–432) ng/mL and 657 (392–1,596) ng/mL for tenofovir and lamivudine, respectively.

### Effects of meningitis on drug exposure in CSF

Seven (50%) individuals had confirmed cryptococcal meningitis by CSF cryptococcal antigen. Plasma and CSF albumin were measured to estimate blood–brain barrier function. CSF to plasma AR was not correlated with postmortem interval (*n* = 16, *r* = −0.13, *P* = 0.6). AR was not correlated with drug exposure in CSF (tenofovir *r* = −0.4, *P* = 0.3; lamivudine *r* = 0.03, *P* = 0.9) or with CSF to plasma ratios (tenofovir *r* = 0.08, *P* = 0.8; lamivudine *r* = 0.11, *P* = 0.7). In addition, there was no relationship between active meningitis and AR (*P* = 0.9).

**Figure 1:**
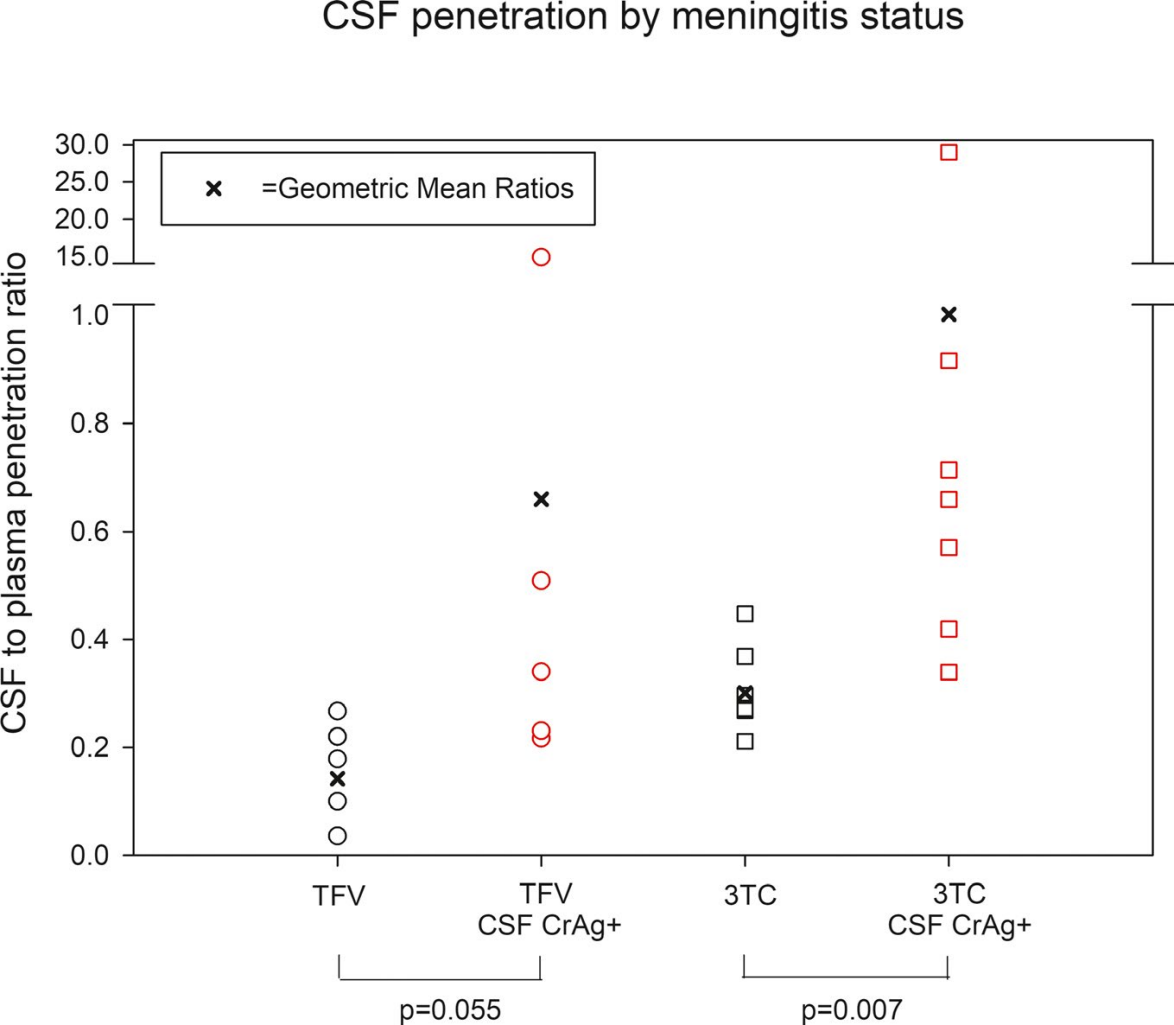
Cerebrospinal fluid (CSF) penetration by meningitis status. CSF to plasma ratio is plotted for tenofovir (TFV) and lamivudine (3TC) by meningitis status as determined by CSF CrAg test. Geometric mean ratios in each group are shown with the dark X. *P* values represent logarithmic paired *t*‐test.

Overall tenofovir CSF to plasma GMR (95% confidence interval) was 0.29 (0.11–0.77). CSF penetration was higher among individuals with cryptococcal meningitis, GMR 0.66 (0.07–5.98) vs. 0.14 (0.06–0.31; **Figure** 1), although this was not statistically significant (*P* = 0.055). Overall, lamivudine CSF to plasma GMR was 0.54 (0.27–1.11). CSF lamivudine penetration was higher among individuals with cryptococcal meningitis, with GMR 1.00 (0.25–4.09) vs. 0.30 (0.24–0.37; *P* = 0.007; **Figure** 1).

### Drug penetration into brain tissue

Brain tissues were collected from four of these individuals, three with cryptococcal meningitis. In this tissue subset, all reported most recent dose within 24 hours prior to death, and tenofovir median concentrations by brain region ranged from 161–2,644 ng/g. Interindividual variability within brain tissue compartments (median percentage of coefficient of variation 84%) was greater than intraindividual variability across brain regions (49%). Mean brain tissue to plasma GMR was 0.36 (0.14–1.24; **Figure** **S1**). Brain tissue to CSF GMR was 0.49 (0.01–5.72).

Median lamivudine concentrations by brain region ranged from 328–784 ng/g. Mean brain tissue to plasma GMR was 0.37 (0.23–0.64; **Figure** **S1**). Although overall variability in brain compartments was less than tenofovir, interindividual variability within brain tissue compartments (median percentage of coefficient of variation 78) was also greater than intraindividual variability across brain regions (27%). Brain tissue to CSF GMR was 0.27 (0.01–1.04).

Median efavirenz concentrations by brain region ranged from 1,227–4,854 ng/g. Mean brain tissue to plasma GMR was 1.28 (1.08–1.79; **Figure** **S1**). Efavirenz tissue concentrations were less variable than tenofovir or lamivudine. Interindividual variability within brain tissue compartments was 51%, whereas intraindividual variability across brain regions was 24%.

## Discussion

To date, knowledge of CNS drug penetration in humans has been limited to CSF. Here, we have described drug exposures in actual brain tissues for three antiretrovirals with widespread global use: tenofovir, lamivudine, and efavirenz. Tenofovir and lamivudine exposures in brain tissue were consistently less than plasma exposures, whereas efavirenz was similar to or slightly higher than plasma exposures. In this small study, there was a consistent trend of lower lamivudine exposure in tissues compared with CSF.

Although we did not measure efavirenz in CSF from our subjects, we observed brain exposures >200‐fold higher than CSF measurements previously reported.^6^ This is similar to predictions by Srinivas *et al*.^7^ whose compartmental pharmacokinetic model predicted efavirenz brain concentrations ranging from 2,300–29,000 ng/mL. Low efavirenz CSF penetration has been attributed to its high (99%) protein binding to plasma proteins. Despite this, high brain penetration has been presumed given the CNS side effects associated with efavirenz use. In this study, we measured total efavirenz and did not assess protein binding in the brain; future studies are needed to understand protein binding in extracellular brain fluid. Protein binding of tenofovir (<7%) and lamivudine (<36%) is low, consistent with their higher CSF to plasma ratios.^8^, ^9^

It is important to interpret our absolute concentrations with caution as no robust studies on postmortem redistribution with antiretrovirals exist. Our observed tenofovir plasma concentrations, and to a lesser extent lamivudine, were higher than previously reported in antemortem subjects.^8^, ^9^ This can, in part, be explained by significant renal impairment in some subjects, as all subjects with creatinine clearance >30 mL/min had concentrations similar to those reported in the package insert.^8^ Creatinine, which is stable in postmortem blood for at least 3 days,^10^ was significantly correlated with tenofovir and lamivudine exposures in our study, but not with efavirenz, consistent with their respective clearance mechanisms. In addition, tenofovir and lamivudine partition into red blood cells and, therefore, drug release upon cell death is likely.^11^ Importantly, the CSF to plasma ratios we observed in patients without meningitis are within the previously reported range suggesting reliability in assessing compartments relative to plasma. Best *et al*.^12^ reported tenofovir CSF concentrations ranging from 0.4–84% (median 5%) of plasma, similar to the 14% we observed. Lamivudine CSF concentrations have previously been reported between 15% and 40% of plasma, consistent with 30% observed in our study.^13^, ^14^

Although inflammation is known to alter the blood–brain barrier, the effect on antiretrovirals has never been quantified. In patients with cryptococcal meningitis, tenofovir penetration into CSF increased from 14% to 66%, and lamivudine penetration increased from 30% to 100%. It is difficult to quantify the effect of inflammation on penetration into brain tissue as three of four individuals had meningitis. Although previous studies have used CSF to plasma AR as a marker for blood–brain barrier function, we did not find AR to be associated with drug penetration, likely due to the small number of subjects in our cohort. One meningitis subject had tenofovir and lamivudine CSF exposures >10‐fold higher than other subjects. The reason for this is unclear, but sensitivity analyses performed excluding this subject did not affect overall results.

There are limitations to this study. First, postmortem drug redistribution is well acknowledged, but how this pertains to antiretrovirals is unknown. However, most studies describing postmortem redistribution pertain to autopsies performed days after death, whereas, in our study, all tissues were collected within 14 hours. Furthermore, CSF and plasma samples collected up to 28 hours postdeath showed no relationship between drug exposure and time between death and autopsy. However, more studies are needed given our small sample size. Second, although our pathology staff has been trained to minimize tissue handling prior to snap freezing, we cannot rule out drug loss during the tissue processing and collection.

In conclusion, these data provide proof of concept for quantitative pharmacology in postmortem tissues as CSF is not a good surrogate for overall drug exposure in CNS. More studies are warranted to elucidate the role of postmortem redistribution and to inform extrapolation from postmortem to antemortem exposures. Antiretroviral brain penetration studies are needed to understand the contribution of drug distribution in establishment of the brain HIV reservoir and to determine the role of inflammation. Understanding drug distribution to and within the CNS will lead to optimal dosing to achieve therapeutic exposure in brain tissue and to more precise dosing in specific patient populations, such as meningitis. In conclusion, postmortem samples provide a unique opportunity to evaluate drug exposure in tissue compartments difficult to sample in human subjects.

## Funding

This work was supported by the University of Minnesota College of Pharmacy, the University of Minnesota Academic Health Center Global Health Seed Grant, the Fogarty International Center (R01NS086312, K01TW010268, and R25TW009345), the National Institute of Neurologic Diseases and Stroke (R01NS086312 and R21NS108344), the National Institute of Allergy and Infectious Diseases (K08AI134262), and the United Kingdom Medical Research Council/Welcome Trust/Department for International Development (MRC MR/M0074131/1).

## Conflicts of Interest

The authors declared no competing interests for this work.

## Author Contributions

M.R.N., K.A.P., J.R., D.A.W., D.B.M., D.R.B., and R.L. wrote the manuscript. M.R.N., K.A.P., J.T., J.R., D.B.M., D.R.B., and R.L. designed the research. M.R.N., K.A.P., J.T., O.C.N., D.A.W., and R.L. performed the research. M.R.N., K.A.P., D.B.M., D.R.B., and R.L. analyzed the data.

**Study Highlights**

**WHAT IS THE CURRENT KNOWLEDGE ON THE TOPIC?**

☑ Antiretrovirals have variable penetration into cerebrospinal fluid (CSF). However, methods to assess brain tissue exposure are limited. Inflammation is known to alter the blood–brain barrier but the effects of meningitis on antiretroviral brain penetration are unknown.

**WHAT QUESTION DID THIS STUDY ADDRESS?**

☑ We sought to compare antiretroviral exposure among plasma, CSF, and brain tissue to identify pockets of brain most vulnerable to inadequate drug exposures. We also examined the effect of meningitis on drug penetration into CSF.

**WHAT DOES THIS STUDY ADD TO OUR KNOWLEDGE?**

☑ Cryptococcal meningitis increased tenofovir and lamivudine penetration into CSF threefold to fivefold. CSF and plasma overpredicted concentrations in brain tissue for tenofovir and lamivudine, whereas efavirenz plasma concentrations were similar to brain concentrations. We provided proof of concept for the use of well‐controlled postmortem tissue collection to quantify tissue exposure.

**HOW MIGHT THIS CHANGE CLINICAL PHARMACOLOGY OR TRANSLATIONAL SCIENCE?**

☑ These data lay the groundwork for future studies using postmortem tissues to characterize drug exposure in compartments difficult to sample.

## Supporting information


**Figure S1.** Variable antiretroviral tissue to plasma ratios across the CNS.

**Table S1.** Subject characteristics.
