# Supplementary material for: Cerebrospinal Fluid and Brain Tissue Penetration of Tenofovir, Lamivudine, and Efavirenz in Postmortem Tissues with Cryptococcal Meningitis
Source: Clin Transl Sci. 2019 Jul 10;12(5):445–9. doi: 10.1111/cts.12661 (PMC6742940; doi:10.1111/cts.12661)
Supplement: Supplementary file 1 — Figure S1. Variable antiretroviral tissue to plasma ratios across the CNS. [file CTS-12-445-s001.pdf]

Figure S1: **Variable antiretroviral tissue to plasma ratios across the CNS**

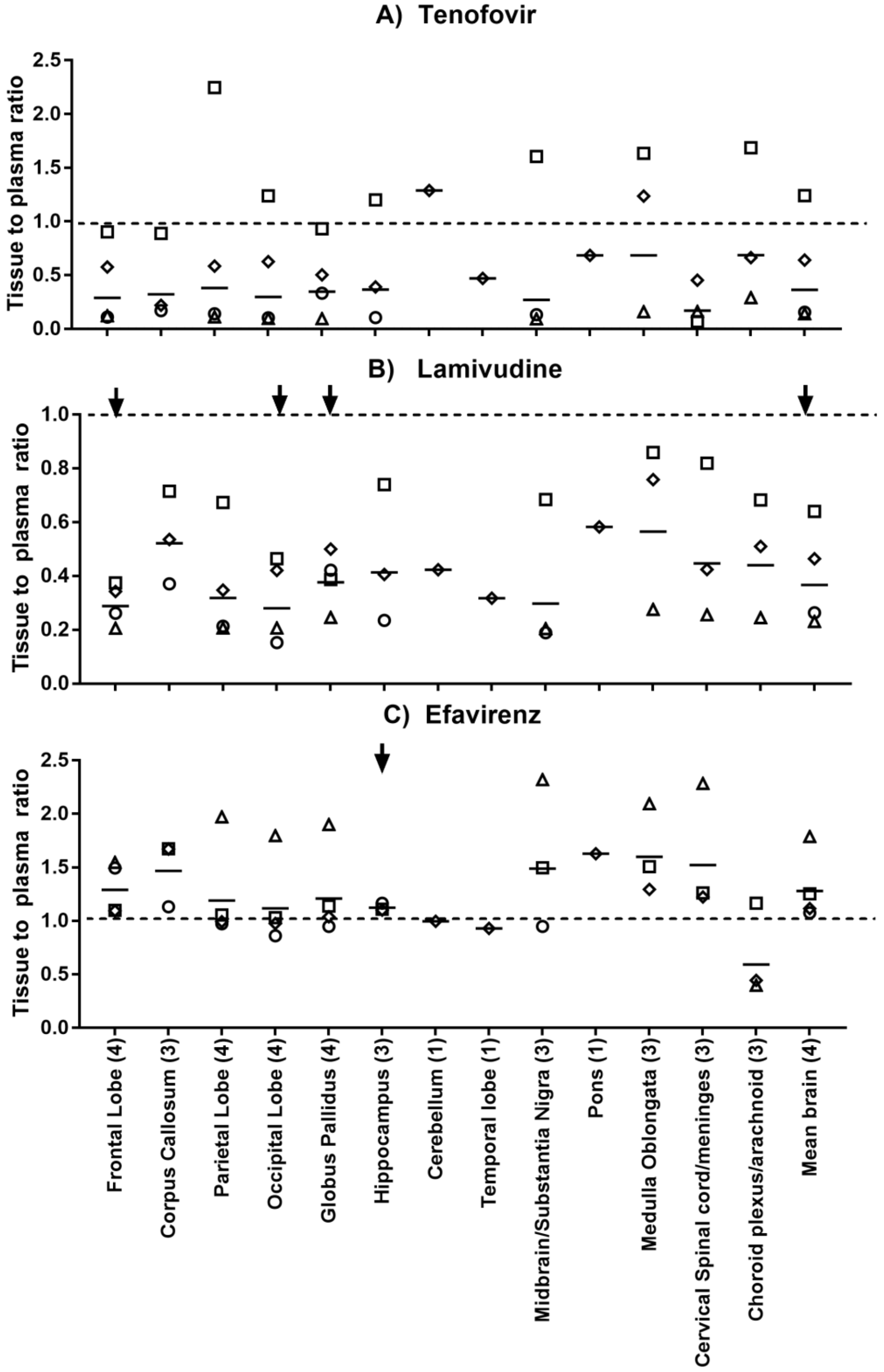

Figure S1 Legend: **Variable antiretroviral tissue to plasma ratios across the CNS**. Brain tissue to plasma ratios are shown for CSF and 13 distinct regions of the brain for A) tenofovir, B) lamivudine, and C) efavirenz. The number of subjects with tissue from each region is in parenthesis on the x-axis. Horizontal bars represent the geometric mean ratio. The dashed line represents a tissue to plasma ratio of 1.00 (tissue exposures = to plasma). Symbols denote different subjects. Subject with CSF CrAg- is denoted with a diamond. Circle, square, and triangle denote subjects with CSF CrAg+. Down arrow ↓indicates $p<0.05$ paired t-test vs plasma, no corrections for multiple comparisons were used.
