# Supplementary material for: Cerebrospinal Fluid and Brain Tissue Penetration of Tenofovir, Lamivudine, and Efavirenz in Postmortem Tissues with Cryptococcal Meningitis
Source: Clin Transl Sci. 2019 Jul 10;12(5):445–9. doi: 10.1111/cts.12661 (PMC6742940; doi:10.1111/cts.12661)
Supplement: Supplementary file 2 — Table S1. Subject characteristics. [file CTS-12-445-s002.pdf]

Table S1: Subject Characteristics

Age, median (range)	39.5 (25-52) years
Sex, n male (%)	10 (63%)
Post-mortem interval, median (range)	5.2 (2.2-28.3) hours
Post-mortem serum creatinine, median (range)	2.3 (0.7-7.3) mg/dL
Antiretroviral regimen (n on each regimen)*	
tenofovir disoproxil fumarate/lamivudine/efavirenz	11
abacavir/lamivudine/atazanavir/ritonavir	2
zidovudine/lamivudine/atazanavir/ritonavir	1
*2 individuals were not on antiretrovirals at time of death but their data were used to assess for post-mortem effects on other laboratory values (i.e. serum creatinine and albumin ratio)	
